# Supplementary material for: Towards unified AI-driven fracture mechanics: the extended deep energy method (XDEM)
Source: Nat Commun. 2026 Aug 15;17:8492. doi: 10.1038/s41467-026-76748-1 (PMC13484471; doi:10.1038/s41467-026-76748-1)
Supplement: Supplementary file 3 [file 41467_2026_76748_MOESM3_ESM.zip › pic/Prerequisite_knowledge/crack.pdf]

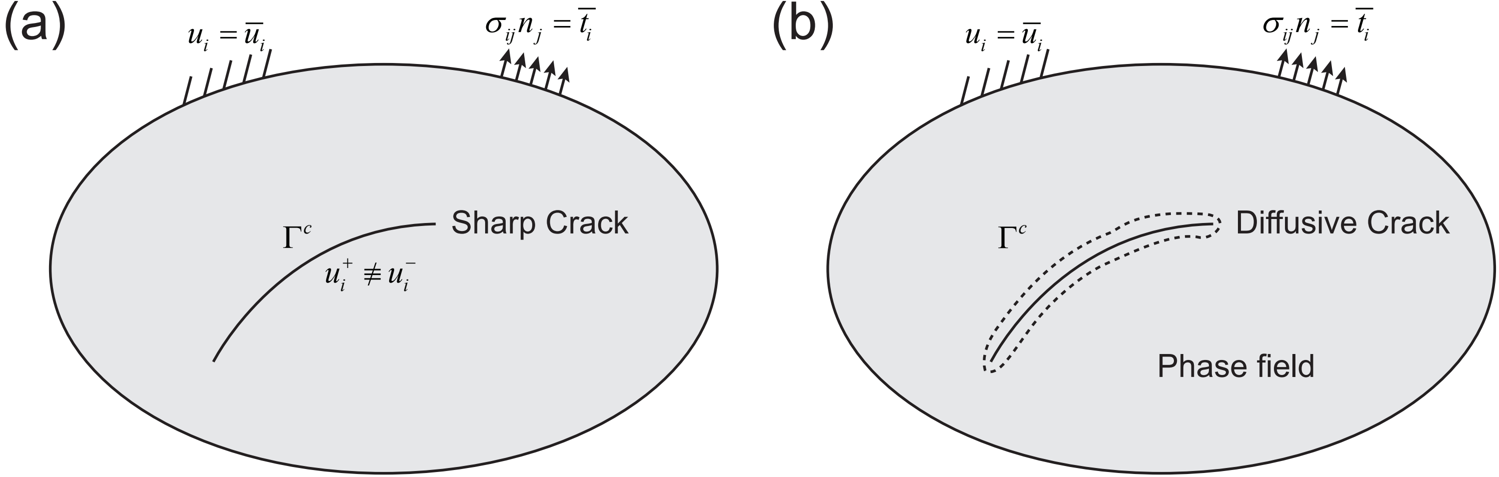
(a)
$u_i = \bar{u}_i$
$\sigma_{ij} n_j = \bar{t}_i$
Γ^c
Sharp Crack
$u_i^+ \not\equiv u_i^-$
(b)
$u_i = \bar{u}_i$
$\sigma_{ij} n_j = \bar{t}_i$
Γ^c
Diffusive Crack
Phase field
